# Mortality after broad‐ versus narrow‐spectrum antibiotic treatment for patients with nursing and healthcare‐associated pneumonia: A nationwide retrospective cohort study

**DOI:** 10.1002/jhm.70195

**Published:** 2025-10-08

**Authors:** Jumpei Taniguchi, Shotaro Aso, Hiroki Matsui, Kiyohide Fushimi, Hideo Yasunaga

**Affiliations:** ^1^ Department of Clinical Epidemiology and Health Economics, School of Public Health The University of Tokyo Tokyo Japan; ^2^ Department of Health Services Research, Graduate School of Medicine The University of Tokyo Tokyo Japan; ^3^ Department of Health Policy and Informatics, Graduate School of Medical and Dental Sciences Institute of Science Tokyo Tokyo Japan

## Abstract

**Background:**

Nursing and healthcare‐associated pneumonia (NHCAP) is defined as pneumonia occurring in individuals with frequent healthcare contact, such as residents of care facilities or patients with impaired activities of daily living. The effectiveness of broad‐spectrum antibiotics in treating NHCAP remains unclear.

**Objective:**

To compare clinical outcomes between broad‐ and narrow‐spectrum antibiotic treatments in patients with NHCAP using a nationwide inpatient database.

**Methods:**

Patients diagnosed with NHCAP between April 2014 and March 2022 were identified from the Diagnosis Procedure Combination inpatient database in Japan. Patients were categorised into those receiving broad‐spectrum antibiotics (antipseudomonal penicillins, antipseudomonal cephalosporins, and carbapenems) and those receiving narrow‐spectrum antibiotics (third‐generation cephalosporins and penicillin plus β‐lactamase inhibitor combinations). Instrumental variable analysis using hospital preference for broad‐spectrum antibiotics was conducted to compare 30‐day in‐hospital mortality. A subgroup analysis was performed for patients with ≥3 risk factors for antibiotic‐resistant pathogens.

**Results:**

Among 828,283 eligible patients, 24.8% received broad‐spectrum antibiotics, while 75.2% received narrow‐spectrum antibiotics. Instrumental variable analysis showed that broad‐spectrum antibiotic use was not associated with 30‐day in‐hospital mortality (10.0% vs. 10.0%; risk difference, 0.0%; 95% confidence interval, −0.7% to 0.8%) compared with narrow‐spectrum antibiotic use. The subgroup analysis of patients with three or more risk factors for antibiotic‐resistant pathogens, broad‐spectrum antibiotic use was also not associated with 30‐day mortality (10.5% vs. 11.0%; risk difference, −0.6%; 95% confidence interval, −2.5% to 1.3%).

**Conclusions:**

Broad‐spectrum antibiotic use was not associated with short‐term in‐hospital mortality in patients with NHCAP, underscoring the importance of individualized antibiotic selection based on patient‐specific risk factors.

## Visual Abstract

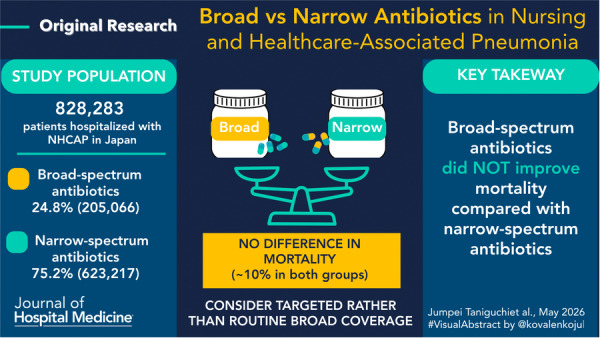

## INTRODUCTION

Nursing and healthcare‐associated pneumonia (NHCAP) is defined as pneumonia occurring in patients with healthcare‐associated risk factors. These include residents of nursing homes; individuals with reduced activities of daily living who are receiving home‐based medical or nursing care; those recently discharged from hospitals; those who have received treatments such as antimicrobial therapy, chemotherapy, immunosuppressive therapy, or dialysis within the past 90 days; and immunocompromised individuals.^1^ The concept of NHCAP was first introduced in the 2011 Japanese Clinical Practice Pneumonia Guidelines, which were developed based on the 2005 American Thoracic Society and Infectious Diseases Society of America guidelines for healthcare‐associated pneumonia (HCAP).^1^, ^2^ However, HCAP was omitted from the American Thoracic Society and Infectious Diseases Society of America pneumonia guidelines in 2019 because HCAP encouraged the overuse of broad‐spectrum antibiotics, which failed to improve the outcomes of patients with HCAP.^3^ The current international guidelines recommend selecting antibiotics based on individual risk factors for drug‐resistant pathogens rather than predefined categories.^3^, ^4^, ^5^

NHCAP continues to be recognised as a distinct category of pneumonia in Japan, owing in large part to an aging population, with a substantial proportion of patients residing in long‐term care facilities or frequent exposure to healthcare settings.^6^, ^7^ The 2024 Japanese pneumonia guidelines reaffirm NHCAP as a distinct entity and acknowledge widespread use of broad‐spectrum antibiotics in clinical practice for patients with risk factors for antibiotic‐resistant pathogens.^6^, ^7^ However, the ability of the NHCAP category to accurately predict the risk of drug‐resistant pathogens is beset by uncertainty. Furthermore, evidence supporting the notion that selecting broad‐spectrum antibiotics based on a diagnosis of NHCAP improves patient outcomes in pneumonia management is lacking. These concerns underscore the need for reassessment of antibiotic selection strategies within the NHCAP framework.

Therefore, this study aimed to evaluate the effect of broad‐spectrum antibiotics on reducing short‐term mortality in patients diagnosed with NHCAP, using a Japanese national inpatient database.

## METHODS

### Ethics

This study received ethical approval from the Institutional Review Board of the University of Tokyo, Japan (3501‐(5), May 19, 2021). As the research was conducted retrospectively using deidentified data, the requirement for obtaining informed consent was waived.

### Data source

This study utilised the Diagnosis Procedure Combination database, a nationwide inpatient database in Japan that integrates both clinical and administrative claims data. It covers data from more than 1200 hospitals across Japan, serving as an important tool for healthcare research.^8^, ^9^ The database contains comprehensive patient information, including demographics (age, sex, height, and weight), primary diagnoses, pre‐existing comorbidities at admission, and complications occurring during hospitalisation. Diagnoses and medical conditions are classified using the International Classification of Diseases, 10th Revision (ICD‐10). Additionally, the database records admission and discharge dates, prescribed medications, surgical interventions, and hospital identifiers. A validation study confirmed its reliability, reporting a diagnostic specificity exceeding 96%, sensitivity ranging from 50% to 80%, and sensitivity and specificity for surgical and procedural records surpassing 90% each.^10^

### Patient selection

We enrolled patients for whom pneumonia was recorded as the diagnosis at admission (ICD‐10 codes: J10.x–J18.x, J69.x) between April 2014 and March 2022 and received either broad‐spectrum or narrow‐spectrum antibiotics within 2 days of hospitalisation. If a patient was hospitalised due to pneumonia multiple times during the study period, only the first admission was included in the analysis. Additionally, participants were required to meet at least one of the following criteria: (i) admission from nursing or other long‐term care facilities; (ii) a history of hospitalisation for conditions other than pneumonia within the past 90 days; (iii) a Barthel Index of 35 or lower at admission (corresponding to a performance status of 3 or higher)^11^; or (iv) a history of treatment with dialysis, antibiotics, chemotherapy, or immunosuppressive therapy within the past 90 days. We excluded the following patients: (i) those who were discharged or died within 2 days of admission; (ii) those who received combination antibiotics therapy with agents other than macrolides, tetracyclines, quinolones, or anti‐methicillin *Staphylococcus aureus* within 2 days of admission; (iii) those who were treated with antifungal or antiviral drugs within 2 days of admission; (iv) those who received both broad‐spectrum and narrow‐spectrum antibiotics simultaneously within 2 days of admission; (v) those with pleural infections (J86.x) or lung abscess (J85.x) at admission; (vi) those who underwent chest tube insertion within 2 days of admission; (vii) those who were transferred from another hospital; (viii) those younger than 15 years; and (ix) pregnant individuals.

### Exposure

Patients were categorized into two groups based on the type of antibiotic: those treated with broad‐spectrum antibiotics (piperacillin/tazobactam, cefepime, ceftazidime, meropenem, imipenem/cilastatin, or doripenem) within 2 days of admission (the broad‐spectrum antibiotics group), and those treated with narrow‐spectrum antibiotics (ceftriaxone, cefotaxime, or ampicillin/sulbactam) within 2 days of admission (the narrow‐spectrum antibiotics group).

### Baseline patient characteristics

Patient characteristics were categorised into three primary groups: patient characteristics at admission; treatments received within 90 days before admission and nutritional intake and treatments received during the first 2 days of hospitalization; and hospital‐related factors. Patient characteristics included variables such as age, sex, body mass index, smoking status (classified as nonsmoker, current smoker, or former smoker), Glasgow Coma Scale score at admission, Barthel Index,^12^ Charlson Comorbidity Index,^13^ pneumonia severity score, risk factors for antibiotic‐resistant pathogens, intensive care or high care unit admission, hospital admission from a nursing home, use of an ambulance, fiscal year, diagnosis of aspiration pneumonia, history of tracheostomy, and comorbid conditions. The comorbidities assessed included hypertension, diabetes mellitus, dyslipidaemia, lung diseases (chronic obstructive pulmonary disease, interstitial pneumonia, bronchiectasis, nontuberculous mycobacterial infections, fungal lung disease, lung cancer, and chronic respiratory failure), oesophageal disorders and dysphagia, cerebrovascular diseases, neurological disorders, cardiovascular diseases, liver diseases, chronic kidney failure, immunodeficiency disorders, haematologic malignancies, nonhaematologic malignancies, history of solid organ transplantation, and dementia (Supporting Information S1: Table 1). Factors pertaining to treatments received within 90 days before admission included prior hospitalisation, use of antibiotics, immunosuppressive therapy, steroid therapy, and chemotherapy with anticancer drugs. Nutritional intake within the first 2 days of hospitalization was classified into oral feeding, tube feeding, or total parenteral nutrition. Interventions received within 2 days of admission encompassed oxygen therapy, mechanical ventilation, vasopressor administration, renal replacement therapy, and the use of tetracyclines, macrolides, quinolones, anti‐methicillin‐resistant *Staphylococcus aureus* agents, corticosteroids, immunosuppressants, proton‐pump inhibitors, hypnotics, and antipsychotics. Hospital‐related characteristics were defined based on whether the facility was a teaching or nonteaching hospital.

Body mass index was categorized into four groups: underweight ( < 18.5 kg/m²), normal weight (18.5–24.9 kg/m²), overweight (25.0–29.9 kg/m²), and obesity ( ≥ 30.0 kg/m²). Functional status was assessed using the Barthel Index, which was divided into four levels: complete dependency (0), severe dependency (5–35), moderate dependency (40–55), mild dependency (60–95), and independency (100). Pneumonia severity was evaluated using the Age, Dehydration, Respiratory Failure, Orientation Disturbance, and Blood Pressure (A‐DROP) scoring system, which is a modified version of the CURB‐65 tool designed by the Japanese Respiratory Society for assessing community‐acquired pneumonia severity; a high A‐DROP score reflects severe pneumonia.^14^, ^15^ The A‐DROP system comprises five clinical indicators, each assigned one point: Age ( ≥ 70 years for men, ≥75 years for women), Dehydration (blood urea nitrogen ≥21 mg/dL or clinical signs of dehydration), Respiratory status (peripheral oxygen saturation ≤90% or partial pressure of oxygen ≤60 Torr), Orientation disturbance (altered mental status), and Blood pressure (systolic blood pressure ≤90 mmHg).^16^ Risk factors for antibiotic‐resistant pathogens were assessed using a composite score, with one point assigned for each of the following criteria: mechanical ventilation within the first 2 days of hospitalization, antibiotic use within 90 days before admission, hospitalisation within the past 90 days before admission, immunocompromised status, tube feeding, underweight (BMI < 18.5 kg/m^2^), and comorbid chronic lung disease.^6^ Immunocompromised status was defined as the presence of immunodeficiency disorders, haematologic malignancies, history of solid organ transplantation, use of chemotherapy within 90 days before admission, or use of immunosuppressive agents, including biologics, disease‐modifying antirheumatic drugs, or corticosteroids within 90 days before admission. Chronic lung disease included chronic obstructive pulmonary disease, interstitial pneumonia, bronchiectasis, nontuberculous mycobacterial infections, fungal lung disease, and lung cancer. Steroid therapy was defined as the administration of corticosteroids at a daily dose equivalent to at least 200 mg of hydrocortisone.

### Outcomes

The primary outcome was 30‐day in‐hospital mortality.

### Statistical analysis

We conducted instrumental variable analysis because important confounding factors such as laboratory test results, imaging findings, and bacterial culture history were not available in our data set. Unmeasured confounders can lead to erroneous inferences regarding the effectiveness of different antibiotic choices. Instrumental variable analysis approximates the random assignment of patients to antibiotic selection, mimicking a randomised clinical trial, even in the presence of unmeasured confounders in the selection of antibiotics for patients with NHCAP. We selected hospital preference for broad‐spectrum antibiotics as the instrumental variable.^17^ The relevance assumption was evaluated by confirming that hospital preference for broad‐spectrum antibiotics was associated with the actual receipt of these antibiotics.^18^ The exclusion restriction assumption was examined by confirming that hospital preference was not directly associated with 30‐day in‐hospital mortality.^18^ Instrumental variable analysis was conducted using a two‐stage residual inclusion framework, adjusting for all baseline characteristics listed in the baseline table as covariates in both stages.^19^ To support the appropriateness of the instrumental variable approach, endogeneity was assessed using first‐stage residuals and evaluated with the Durbin–Wu–Hausman test.

### Subgroup analysis

Since the 2024 pneumonia treatment guidelines recommend the use of broad‐spectrum antibiotics for all NHCAP patients with three or more risk factors for antibiotic‐resistant pathogens, we performed subgroup analysis stratified by the presence of three risk factors.^6^

### Sensitivity analysis

We conducted several sensitivity analyses to assess the robustness of our findings. First, we conducted an analysis that excluded patients who received combination therapy with quinolones to consider that some patients in the narrow‐spectrum group may have received levofloxacin for *Pseudomonas aeruginosa* coverage. Second, we restricted the analysis to patients who were admitted from nursing or other long‐term care facilities to evaluate the utility of broad‐spectrum antibiotic use in this subset. Third, we performed a more conservative analysis by excluding underweight as a risk factor for antibiotic‐resistant pathogens, considering that we used underweight as a risk factor instead of hypoalbuminaemia.

Continuous variables were analysed using *t* tests and expressed as means with standard deviations, while categorical variables were assessed with *χ* ^2^ tests and reported as frequencies with percentages. Two‐tailed *p* valued less than .05 were considered statistically significant. All analyses were conducted using STATA/SE version 18.0 (StataCorp).

## RESULTS

**Figure 1:**
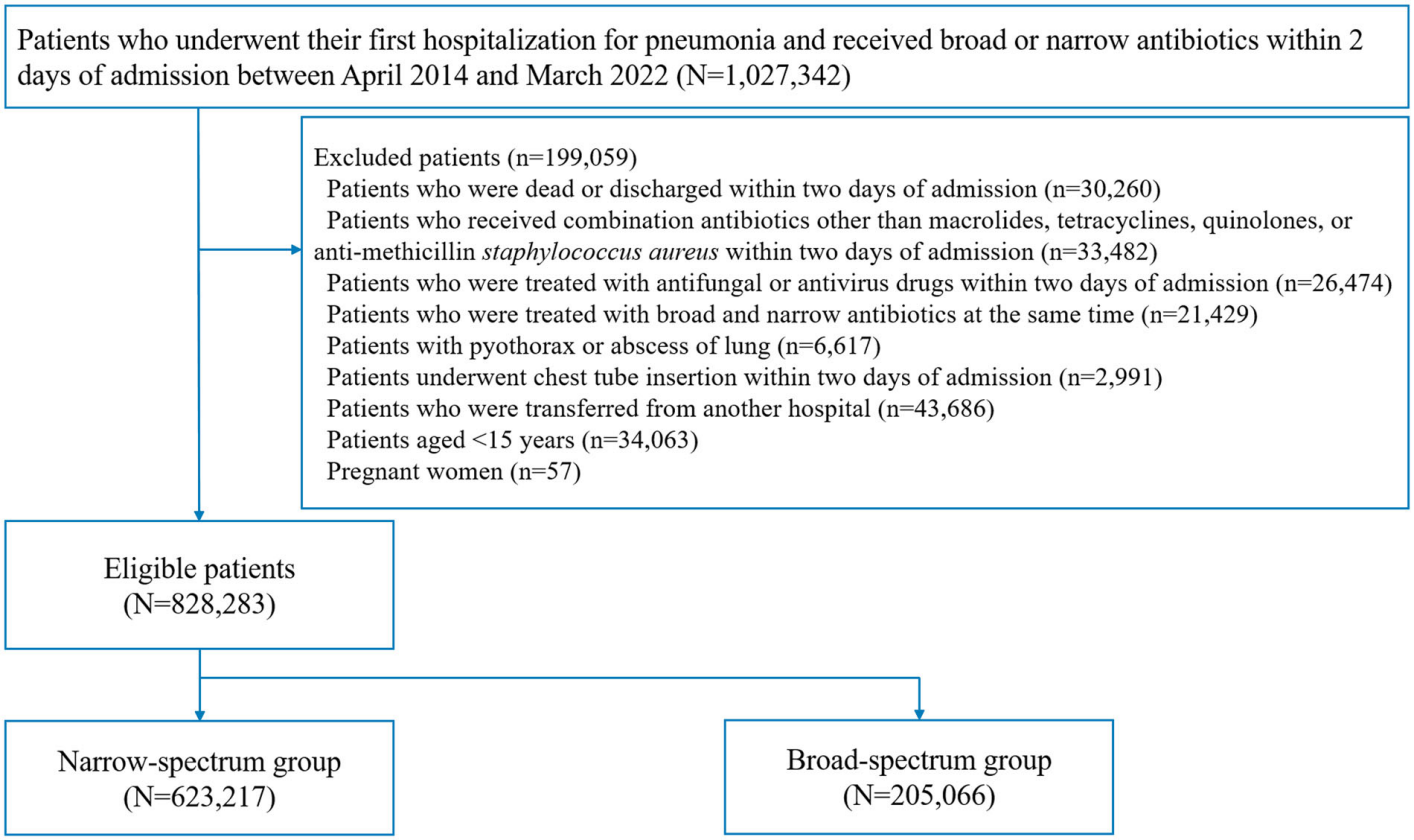
Study design.

We identified 1,027,342, patients who were hospitalised due to NHCAP and received broad‐ or narrow‐spectrum antibiotics between April 2014 and March 2022 (Figure 1). A total of 828,283 patients were eligible. Among patients who met the criteria of NHCAP, 38.7% were admitted from a nursing or other long‐term care facility, 16.7% had a history of hospitalisation within the past 90 days, 79.4% had a performance status of three or higher at admission (corresponding to a Barthel Index of 35 or lower), and 26.1% had received dialysis, antibiotics, chemotherapy, or immunosuppressive therapy within the past 90 days. Overall, 24.8% (205,066/828,283) of patients received broad‐spectrum antibiotics and 75.2% (623,217/828,283) received narrow‐spectrum antibiotics. In the broad‐spectrum group, 72.6% of patients received antipseudomonal penicillin (piperacillin/tazobactam), 6.0% received antipseudomonal cephalosporins (cefepime, ceftazidime), and 21.5% received carbapenems (meropenem, imipenem/cilastatin, doripenem). In the narrow‐spectrum group, 33.0% received third‐generation cephalosporins (ceftriaxone, cefotaxime), and 67.0% received a combination of a penicillin and β‐lactamase inhibitor (ampicillin/sulbactam). The mean duration of antibiotic administration was 8.8 days (standard deviation, 4.3) in the broad‐spectrum group and 8.2 days (standard deviation, 4.1) in the narrow‐spectrum group.

Table 1 shows the baseline characteristics. Patients in the broad‐spectrum antibiotic group were more likely to be younger and had higher Barthel Index values. Pneumonia severity scores and the frequency of risk factors for antibiotic‐resistant pathogens were higher in the broad‐spectrum antibiotic group than those in the narrow‐spectrum antibiotic group. Patients in the broad‐spectrum antibiotic group were more likely to receive treatments such as mechanical ventilation, vasopressors, anti‐methicillin‐resistance *Staphylococcus aureus* agents, and steroids within the first 2 days of hospitalization. In the unadjusted population, 30‐day in‐hospital mortality was higher in the broad‐spectrum group than that in the narrow‐spectrum group [15.3% vs. 10.0%; risk difference (RD), 5.2%; 95% confidence interval (CI), 5.1% to 5.4%; *p* < .001].

**Table 1:** Patient characteristics.

Variables	Narrow‐spectrum group (*n* = 623,217)	Broad‐spectrum group (*n* = 205,066)	Standardised difference (%)
Age, years, mean (SD)	83.3 (11.2)	81.4 (11.3)	−16.1
Male, *n* (%)	326,173 (52.3)	120,804 (58.9)	13.3
BMI, kg/m^2^, *n* (%)			
<18.50	224,573 (36.0)	76,778 (37.4)	2.9
18.50–24.99	261,413 (41.9)	87,233 (42.5)	1.2
25.00–29.99	41,699 (6.7)	13,346 (6.5)	−0.7
≥30.00	7786 (1.2)	2416 (1.2)	−0.6
Missing data	87,746 (14.1)	25,293 (12.3)	−5.2
Smoking history, *n* (%)			
Nonsmoker	413,315 (66.4)	126,600 (61.7)	−9.6
Current/past smoker	125,261 (20.1)	50,660 (24.7)	11.1
Missing data	84,441 (13.5)	27,806 (13.6)	0.0
GCS on admission, mean (SD)	13.9 (2.2)	13.7 (2.4)	−6.1
Barthel index on admission, *n* (%)			
0	357,624 (57.4)	115,904 (56.5)	−1.7
5–35	144,423 (23.2)	39,412 (19.2)	−9.7
40–55	23,085 (3.7)	7655 (3.7)	0.2
60–95	20,218 (3.2)	8912 (4.3)	5.8
100	35,576 (5.7)	17,645 (8.6)	11.3
Missing data	42,291 (6.8)	15,538 (7.6)	3.1
Charlson comorbidity index, mean (SD)	1.5 (1.6)	1.6 (1.6)	8.1
Pneumonia severity score, mean (SD)	2.1 (1.1)	2.3 (1.2)	15.7
Risk factors for antibiotic‐resistant pathogens, *n* (%)			
0	276,677 (44.4)	70,546 (34.4)	−20.6
1–2	296,756 (47.6)	102,158 (49.8)	4.4
≥3	49,784 (8.0)	32,362 (15.8)	24.3
ICU admission, *n* (%)	6056 (1.0)	5112 (2.5)	11.7
HCU admission, *n* (%)	23,610 (3.8)	11,123 (5.4)	7.8
Admission from nursing home, *n* (%)	245,418 (39.4)	75,421 (36.8)	−5.4
Ambulance transport, *n* (%)	337,589 (54.2)	111,365 (54.3)	0.3
Fiscal year, *n* (%)			
2014–2015	148,983 (23.9)	50,309 (24.5)	1.5
2016–2017	156,412 (25.1)	49,203 (24.0)	4.6
2018–2019	143,943 (23.1)	46,805 (22.8)	−0.6
2020–2021	173,879 (27.9)	58,749 (28.6)	1.7
Aspiration pneumonia, *n* (%)	335,390 (53.8)	93,845 (45.8)	−16.2
History of tracheostomy, *n* (%)	588 (0.1)	266 (0.1)	1.1
Hypertension, *n* (%)	178,778 (28.7)	50,368 (24.6)	−9.3
Diabetes mellitus, *n* (%)	102,167 (16.4)	35,963 (17.5)	3.0
Dyslipidaemia, *n* (%)	43,609 (7.0)	12,100 (5.9)	−4.5
Lung disease, *n* (%)			
Chronic obstructive pulmonary disease	34,044 (5.5)	14,881 (7.3)	7.4
Interstitial pneumonia	12,255 (2.0)	8671 (4.2)	13.1
Bronchiectasis & NTM of the lungs	4929 (0.8)	3444 (1.7)	8.1
Fungal lung disease	991 (0.2)	1003 (0.5)	5.8
Lung cancers	18,084 (2.9)	11,373 (5.5)	13.2
Chronic respiratory failure	10,037 (1.6)	5662 (2.8)	7.9
Oesophageal disorders and dysphagia, *n* (%)	7312 (4.2)	10,867 (3.6)	−3.1
Cerebrovascular disease, *n* (%)	121,267 (19.5)	33,839 (16.5)	−7.7
Neurologic disease, *n* (%)	32,645 (5.2)	8986 (4.4)	−4.0
Cardiovascular disease, *n* (%)	121,880 (19.6)	40,135 (19.6)	0.0
Liver disease, *n* (%)	15,207 (2.4)	5850 (2.9)	2.6
Chronic kidney failure, *n* (%)	35,287 (5.7)	13,459 (6.6)	3.8
Immunodeficiency disorders, *n* (%)	243 (0.0)	264 (0.1)	3.1
Haematological malignancy, *n* (%)	4359 (0.7)	4234 (2.1)	11.7
Nonhaematological malignancy, *n* (%)	49,970 (8.0)	22,206 (10.8)	9.6
Solid organ transplantation, *n* (%)	367 (0.1)	375 (0.2)	3.6
Dementia, *n* (%)	161,339 (25.9)	41,838 (20.4)	−13.0
Factors before 90 days of admission, *n* (%)			
Hospitalisation	94,655 (15.2)	43,629 (21.3)	15.8
Antibiotic use	77,190 (12.4)	39,628 (19.3)	19.1
Immunosuppressive therapy	4165 (0.7)	3332 (1.6)	9.0
Steroid	32,464 (5.2)	22,738 (11.1)	21.6
Chemotherapy	15,899 (2.6)	14,082 (6.9)	20.5
Nutrition within 2 days of admission, *n* (%)			
Oral feeding	319,478 (51.3)	92,275 (45.0)	−12.6
Tube feeding	16,372 (2.6)	7638 (3.7)	6.3
Total parenteral nutrition	5075 (0.8)	2195 (1.1)	2.7
Treatment within 2 days of admission, *n* (%)			
Oxygenation	392,555 (63.0)	137,631 (67.1)	8.7
Mechanical ventilation	18,245 (2.9)	14,652 (7.1)	19.4
Vasopressors	12,987 (2.1)	13,912 (6.8)	23.0
Renal replacement therapy	6883 (1.1)	3454 (1.7)	4.9
Tetracyclines	4226 (0.7)	2233 (1.1)	4.4
Macrolides	28,041 (4.5)	12,409 (6.1)	6.9
Quinolones	8109 (1.3)	6491 (3.2)	12.6
Anti‐MRSA antibiotics	1450 (0.2)	3071 (1.5)	13.7
Steroids	14,677 (2.4)	10,165 (5.0)	13.9
Immunosuppressants	2541 (0.4)	1713 (0.8)	5.4
Proton pump inhibitors	130,530 (20.9)	57,788 (28.2)	16.9
Hypnotics	94,066 (15.1)	33,571 (16.4)	3.5
Antipsychotics	72,175 (11.6)	22,067 (10.8)	−2.6
Teaching hospital admission, *n* (%)	477,274 (76.6)	149,662 (73.0)	−8.3

*Note*: The total number of aetiologies does not add up to 100% as more than one cause can be assigned to a single patient.

Abbreviations: BMI, body mass index; GCS, Glasgow Coma Scale; HCU, high care unit; ICU, intensive care unit; MRSA, methicillin‐resistant *Staphylococcus aureus*; NTM, nontuberculosis mycobacterium; SD, standard deviation.

### Main analysis

Patient characteristics across hospital preference and the estimation results from both stages of the model are presented in Supporting Information S1: Tables 2–4. Hospital preference for broad‐spectrum antibiotics was strongly associated with the actual receipt of broad‐spectrum antibiotics (*F* (1, 308143) = 30,474). Hospital preference was not associated with 30‐day in‐hospital mortality (Supporting Information S1: Table 2). The residual from the first‐stage regression was statistically significant in the second‐stage model (odds ratio: 1.29; 95% CI, 1.17–1.42; *p* < .001), and the Durbin–Wu–Hausman test also indicated endogeneity (*χ*² = 26.37, *p* < .001), supporting the use of the instrumental variable approach (Supporting Information S1: Tables 3–4).

Instrumental variable analysis found no significant difference in 30‐day in‐hospital mortality between the broad‐spectrum and narrow‐spectrum antibiotic groups (10.0% vs. 10.0%; RD, 0.0%; 95% CI, −0.7% to 0.8%; *p* = .900) (Table 2).

**Table 2:** Comparison of outcomes between the groups.

Primary outcome (%)	Narrow‐spectrum group	Broad‐spectrum group	Risk difference	95% confidence interval	*p*
30 day in‐hospital mortality	10.0%	10.0%	0.0%	−0.7% to 0.8%	0.900

### Subgroup analysis

**Figure 2:**
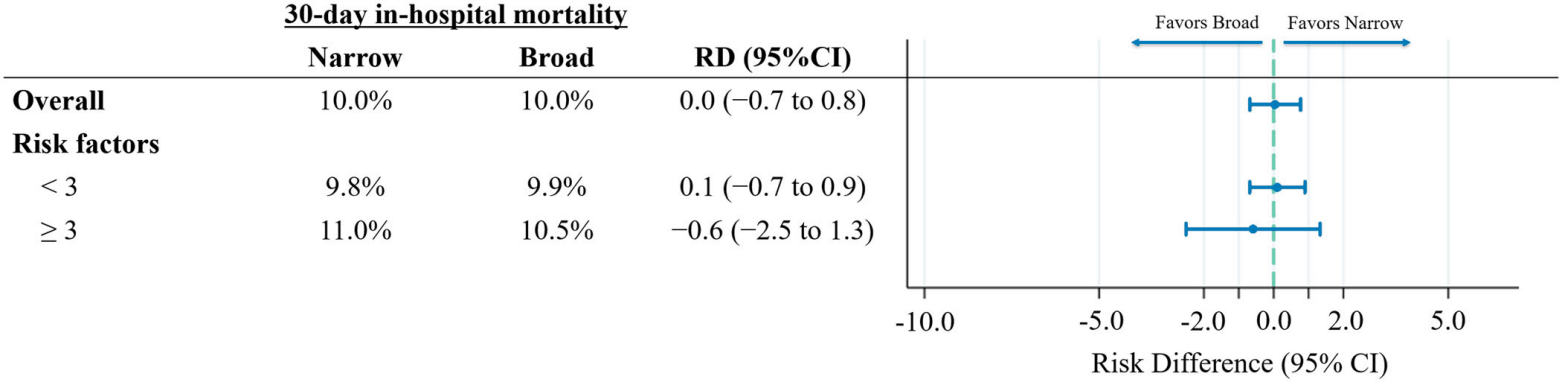
Subgroup analysis for 30‐day in‐hospital mortality between the narrow‐ and broad‐spectrum antibiotic groups. CI, confidence interval; RD, risk difference.

The results of the subgroup analysis are shown in Figure 2. Among patients possessing more than three risk factors for antibiotic‐resistant pathogens, broad‐spectrum antibiotic use was not associated with 30‐day in‐hospital mortality compared with narrow‐spectrum antibiotic use (10.5% vs. 11.0%; RD, −0.6%; 95% CI, −2.5% to 1.3%; *p* = .547) (Figure 2).

The results of the sensitivity analyses were consistent with those of the main analysis (Supporting Information S1: Table 5).

## DISCUSSION

In this retrospective cohort study, we evaluated and compared the effect of broad‐spectrum antibiotics with that of narrow‐spectrum antibiotics on 30‐day in‐hospital mortality in patients with NHCAP. Our findings showed that the use of broad‐spectrum antibiotics was not associated with 30‐day in‐hospital mortality in patients with NHCAP. Similarly, among patients stratified by the presence of three or more risk factors for antibiotic‐resistant pathogens, broad‐spectrum antibiotic administration was not associated with 30‐day in‐hospital mortality. These results suggest that selecting broad‐spectrum antibiotics for NHCAP may not improve short‐term in‐hospital mortality.

Two previous small randomised controlled trials conducted primarily in nursing‐home residents demonstrated that the use of broad‐spectrum antibiotics did not improve short‐term clinical outcomes in pneumonia.^20^, ^21^ The sample size of these studies was small and the blinding design was insufficient. Additionally, a small single‐centre, prospective observational study of patients with NHCAP found no significant association between broad‐spectrum antibiotic use and improved outcomes.^22^ Concerns about selection bias existed because it is possible that patients with more severe conditions were preferentially treated with broad‐spectrum antibiotics. Previous large‐scale cohort and administrative database studies involving patients with HCAP also reported that broad‐spectrum antibiotics did not improve short‐term outcomes.^23^, ^24^ Our findings suggest that selecting broad‐spectrum antibiotics for NHCAP may not improve patient outcomes, and resemble the observations in HCAP.

There may be an explanation for our results. Akin to HCAP, the definition of NHCAP may not reliably identify patients with antibiotic‐resistant pathogens. Previous studies have demonstrated that the HCAP category did not consistently predict a high prevalence of antibiotic‐resistant pathogens.^25^ Furthermore, the application of the HCAP classification was associated with increased use of broad‐spectrum antibiotics without corresponding improvements in clinical outcomes.^23^, ^24^, ^25^, ^26^ Because the concept of NHCAP is rooted in HCAP and both entities employ similar criteria, the NHCAP category itself may also be beset by limitations in predicting antibiotic‐resistant pathogens, even in settings with aging populations and frequent healthcare exposure such as Japan. Indeed, *Pseudomonas aeruginosa* has been detected in approximately 5% of NHCAP cases.^6^, ^27^, ^28^ The current definition of NHCAP may include factors possessing suboptimal predictive accuracy. Because the NHCAP classification may retain clinical utility by identifying a population more vulnerable than typical community‐acquired pneumonia, it can support prognostic assessment and risk communication with patients and their families. However, antibiotic selection for NHCAP would ideally be guided by an individualized approach based on validated risk factors—such as prior isolation of resistant organisms, recent hospitalization, or exposure to parenteral antibiotics—as well as local antimicrobial susceptibility patterns. Consistent with standard community‐acquired pneumonia management practices, initial empiric broad‐spectrum coverage may be appropriate in severe cases of NHCAP, but prompt de‐escalation should be considered once microbiological data become available.

The major strengths of our study are the use of a large, nationwide database and the application of instrumental variable analysis. However, this study has some limitations. First, we could not obtain clinically important information including patient medical history, laboratory data, imaging findings, and bacterial culture results. Recent bacterial detection history plays a crucial role in the selection of broad‐spectrum antibiotics. Additionally, selection bias may have led to the use of broad‐spectrum antibiotics in patients with more severe conditions who received more intensive treatment. Therefore, in this study, we used instrumental variable analysis to account for these unmeasured confounders. Second, although the instrumental variable met the core assumptions—being strongly associated with treatment assignment and not directly linked to outcomes—it cannot fully eliminate the risk of bias from unmeasured confounding. Furthermore, the resulting estimates reflect the local average treatment effect, which pertains only to patients whose treatment assignment was influenced by the instrument. Thus, these findings may not be generalizable to the entire NHCAP population. Although we confirmed the robustness of our findings through subgroup and sensitivity analyses, future studies incorporating more detailed clinical information or employing prospective study designs will be warranted to confirm our results. Third, although this study included a large‐scale database comprising data from over 1200 hospitals across Japan, the data were limited to a single country and primarily represent an Asian population. The definition of pneumonia in this study, which includes patients in nursing homes, long‐term care facilities, and those with frequent healthcare exposure, provides valuable insights into healthcare provision in an aging population. However, the generalisability of NHCAP, as defined by the Japanese Respiratory Society, may be limited to other countries and regions with different healthcare systems and epidemiological settings.

## CONCLUSION

The use of broad‐spectrum antibiotics for patients with NHCAP was not associated with 30‐day in‐hospital mortality compared with narrow‐spectrum antibiotics. Our findings support the importance of tailoring antibiotic therapy to individual risk factors for drug‐resistant pathogens in patients with NHCAP.

## CONFLICT OF INTEREST STATEMENT

The authors declare no conflict of interest.

## ETHICS STATEMENT

This study was approved by the Institutional Review Board of the University of Tokyo, which waived the need for informed consent owing to the retrospective design. The Institutional Review Board waived the requirement for informed consent because of the anonymous nature of the data.

## Supporting information

Supporting information.

## Data Availability

The datasets are not available publicly.
