# Supplementary material for: Mortality after broad‐ versus narrow‐spectrum antibiotic treatment for patients with nursing and healthcare‐associated pneumonia: A nationwide retrospective cohort study
Source: J Hosp Med. 2025 Oct 8;21(5):477–86. doi: 10.1002/jhm.70195 (PMC13136921; doi:10.1002/jhm.70195)
Supplement: Supplementary file 1 — Supporting information. [file JHM-21-477-s001.docx]

**Supplementary Table 1**. **ICD-10 codes used to define each comorbidity**

| Tracheostomy | J95.0, Z43.0, Z93.0 |
| --- | --- |
| Hypertension | I10.x, I11.0, I11.9 |
| Diabetes mellitus | E10.x–E14.x |
| Dyslipidaemia | E78.x |
| Chronic obstructive pulmonary disease | J43.x, J44.x |
| Interstitial Pneumonia | J84.x, J99.x |
| Bronchiectasis and NTM of the lungs | A31.0, J47.x |
| Fungal lung disease | B37.1, B38.0, B38.1, B38.2, B39.0, B39.1, B39.2, B40.0, B40.1, B40.2, B41.0, B42.0, B44.0, B44.1, B45.0, B46.0 |
| Lung cancer | C34.x |
| Chronic respiratory failure | J96.1 |
| Oesophageal disorders and dysphagia | C15.x, F45.3, J39.8, J95.0, J98.0, K11.7, K22.0, K22.2, K22.5, K22.8, K44.9, R13.x, R19.8 |
| Cerebrovascular disease | I60.x–I69.x |
| Neurologic disease | G12.1, G12.2, G20.x, G35.x, G70.0 |
| Cardiovascular disease | I20.x–25.x, I42.x, I50.x |
| Liver disease | B16.x–19.x, K70.x–77.x |
| Chronic kidney failure | I12.0, N18.x–19.x |
| Immunodeficiency disorders | B20.x–B24.x, D80.x–D84.x, Z21.x |
| Haematologic malignancy | C81.x–96.x, T86.0, T86.8, T86.9, Y83.8, Z94.8, Z94.9 |
| Non-haematologic malignancy | C00.x–14.x, C16.x–C22.x, C25.x–C32.x, C35.x–C80.x, C97.x, D00.x–09.x |
| Solid organ transplantation | T86.1, T86.2, T86.3, T86.4, Z94.x |
| Dementia | F00.x–F03.x, G30.x |

NTM, non-tuberculosis mycobacterium, ICD-10: International Classification of Diseases, 10^th^ Revision

**Supplementary Table 2. Patient characteristics by median hospital preference**

| Variables | Hospital preference for broad-spectrum antibiotics < 22.9  (n = 414,125) | Hospital preference for broad-spectrum antibiotics ≥ 22.9  (n = 414,128) | Standardised difference  (%) |
| --- | --- | --- | --- |
| Age, years, mean (SD) | 83.7 (10.6) | 81.9 (11.8) | −15.3 |
| Male, n (%) | 217,002 (52.4) | 229,975 (55.5) | 6.3 |
| BMI, kg/m^2^, n (%) |  |  |  |
| <18.50 | 155,073 (37.4) | 146,278 (35.3) | −4.4 |
| 18.50–24.99 | 170,410 (41.1) | 178,236 (43.0) | 3.8 |
| 25.00–29.99 | 25,820 (6.2) | 29,225 (7.1) | 3.3 |
| ≥30.00 | 4,624 (1.1) | 5,578 (1.3) | 2.1 |
| Missing data | 58,198 (14.1) | 54,841 (13.2) | −2.4 |
| Smoking history, n (%) |  |  |  |
| Nonsmoker | 274,766 (66.3) | 265,349 (64.1) | −4.8 |
| Current/past smoker | 80,823 (19.5) | 95,098 (23.0) | 8.4 |
| Missing data | 58,536 (14.1) | 53,711 (13.0) | −3.4 |
| GCS on admission, mean (SD) | 13.8 (2.2) | 13.9 (2.3) | 2.7 |
| Barthel index on admission, n (%) |  |  |  |
| 0 | 246,443 (59.5) | 227,085 (54.8) | −9.5 |
| 5–35 | 93,875 (22.7) | 89,960 (21.7) | −2.3 |
| 40–55 | 15,044 (3.6) | 15,696 (3.8) | 0.8 |
| 60–95 | 11,790 (2.8) | 17,340 (4.2) | 7.3 |
| 100 | 19,439 (4.7) | 33,782 (8.2) | 14.2 |
| Missing data | 27,534 (6.6) | 30,295 (7.3) | 2.6 |
| Charlson comorbidity index, mean (SD) | 1.5 (1.6) | 1.5 (1.6) | 3.2 |
| Pneumonia severity score, mean (SD) | 2.1 (1.1) | 2.1 (1.1) | −1.8 |
| Risk factors for antibiotic-resistant pathogens, n (%) |  |  |  |
| 0 | 181,279 (43.8) | 165,944 (40.1) | −7.5 |
| 1–2 | 199,749 (48.2) | 199,165 (48.1) | −0.3 |
| ≥3 | 33,097 (8.0) | 49,049 (11.8) | 12.9 |
| ICU admission, n (%) | 5,034 (1.2) | 6,134 (1.5) | 2.3 |
| HCU admission, n (%) | 18,956 (4.6) | 15,777 (3.8) | −3.8 |
| Admission from nursing home, n (%) | 173,226 (41.8) | 147,613 (35.6) | −12.7 |
| Ambulance transport, n (%) | 225,321 (54.4) | 223,633 (54.0) | −0.8 |
| Fiscal year, n (%) |  |  |  |
| 2014–2015 | 104,368 (25.2) | 94,924 (22.9) | −5.3 |
| 2016–2017 | 103,329 (25.0) | 102,286 (24.7) | −0.6 |
| 2018–2019 | 94,113 (22.7) | 96,635 (23.3) | 1.4 |
| 2020–2021 | 112,315 (27.1) | 120,313 (29.1) | 4.3 |
| Aspiration pneumonia, n (%) | 226,951 (54.8) | 202,284 (48.8) | −11.9 |
| History of tracheostomy, n (%) | 398 (0.1) | 456 (0.1) | 0.4 |
| Hypertension, n (%) | 120,921 (29.2) | 108,235 (26.1) | −6.9 |
| Diabetes mellitus, n (%) | 68,205 (16.5) | 69,925 (16.9) | 1.1 |
| Dyslipidaemia, n (%) | 28,934 (7.0) | 26,775 (6.5) | −2.1 |
| Lung disease, n (%) |  |  |  |
| Chronic obstructive pulmonary disease | 24,187 (5.8) | 24,738 (6.0) | 0.6 |
| Interstitial Pneumonia | 8,826 (2.1) | 12,100 (2.9) | 5.0 |
| Bronchiectasis & NTM of the lungs | 3,702 (0.9) | 4,671 (1.1) | 2.3 |
| Fungal lung disease | 735 (0.2) | 1,259 (0.3) | 2.6 |
| Lung cancers | 11,312 (2.7) | 18,145 (4.4) | 8.9 |
| Chronic respiratory failure | 7,009 (1.7) | 8,690 (2.1) | 3.0 |
| Oesophageal disorders and dysphagia, n (%) | 33,877 (8.2) | 31,076 (7.5) | −2.5 |
| Cerebrovascular disease, n (%) | 85,259 (20.6) | 69,847 (16.9) | −9.6 |
| Neurologic disease, n (%) | 21,396 (5.2) | 20,235 (4.9) | −1.3 |
| Cardiovascular disease, n (%) | 82,951 (20.0) | 79,064 (19.1) | −2.4 |
| Liver disease, n (%) | 9,982 (2.4) | 11,075 (2.7) | 1.7 |
| Chronic kidney failure, n (%) | 23,065 (5.6) | 25,681 (6.2) | 2.7 |
| Immunodeficiency disorders, n (%) | 110 (0.0) | 397 (0.1) | 2.8 |
| Haematological malignancy, n (%) | 3,164 (0.8) | 5,429 (1.3) | 5.4 |
| Non-haematological malignancy, n (%) | 31,095 (7.5) | 41,081 (9.9) | 8.6 |
| Solid organ transplantation, n (%) | 87 (0.0) | 655 (0.2) | 4.6 |
| Dementia, n (%) | 110,489 (26.7) | 92,688 (22.4) | −10.0 |
| Factors prior to 90 days of admission, n (%) |  |  |  |
| Hospitalisation | 62,679 (15.1) | 75,065 (18.1) | 8.0 |
| Antibiotic use | 48,000 (11.6) | 68,818 (16.6) | 14.5 |
| Immunosuppressive therapy | 1,995 (0.5) | 5,502 (1.3) | 8.9 |
| Steroid | 19,094 (4.6) | 36,108 (8.7) | 16.5 |
| Chemotherapy | 8,909 (2.2) | 21,072 (5.1) | 15.8 |
| Nutrition within 2 days of admission, n (%) |  |  |  |
| Oral feeding | 207,287 (50.1) | 204,466 (49.4) | −1.4 |
| Tube feeding | 10,871 (2.6) | 13,139 (3.2) | 3.3 |
| Total parenteral nutrition | 3,607 (0.9) | 3,663 (0.9) | 0.1 |
| Treatment within 2 days of admission, n (%) |  |  |  |
| Oxygenation | 263,676 (63.7) | 266,510 (64.3) | 1.4 |
| Mechanical ventilation | 13,933 (3.4) | 18,964 (4.6) | 6.2 |
| Vasopressors | 11,503 (2.8) | 15,396 (3.7) | 5.3 |
| Renal replacement therapy | 4,555 (1.1) | 5,782 (1.4) | 2.7 |
| Tetracyclines | 3,043 (0.7) | 3,416 (0.8) | 1.0 |
| Macrolides | 16,673 (4.0) | 23,777 (5.7) | 8.0 |
| Quinolones | 5,402 (1.3) | 9,198 (2.2) | 7.0 |
| Anti-MRSA antibiotics | 1,729 (0.4) | 2,792 (0.7) | 3.5 |
| Steroids | 12,005 (2.9) | 12,837 (3.1) | 1.2 |
| Immunosuppressants | 1,396 (0.3) | 2,858 (0.7) | 4.9 |
| Proton pump inhibitors | 80,327 (19.4) | 107,991 (26.1) | 16.0 |
| Hypnotics | 57,315 (13.8) | 70,322 (17.0) | 8.7 |
| Antipsychotics | 44,748 (10.8) | 49,494 (12.0) | 3.6 |
| Teaching hospital admission, n (%) | 315,288 (76.1) | 311,648 (75.2) | −2.1 |
| 30-day in hospital death, n (%) | 45,782 (11.1) | 47,920 (11.6) | 1.6 |

BMI, body mass index; GCS, Glasgow Coma Scale; HCU, high care unit; ICU, intensive care unit; NTM, non-tuberculosis mycobacterium; MRSA, methicillin-resistant *Staphylococcus aureus*; SD, standard deviation.

**Supplementary Table 3. First-stage regression results**

| Variables | Odds ratio  (95% Confidence interval) | *P* |
| --- | --- | --- |
| Hospital preference | 149.10 (140.20–158.56) | < 0.001 |
| Age | 0.99 (0.99–0.99) | < 0.001 |
| Male | 1.14 (1.12–1.16) | < 0.001 |
| BMI, kg/m^2^ |  |  |
| <18.50 | 1.04 (1.01–1.08) | 0.008 |
| 18.50–24.99 | Reference |  |
| 25.00–29.99 | 0.89 (0.86–0.92) | < 0.001 |
| ≥30.00 | 0.81 (0.75–0.86) | < 0.001 |
| Smoking history |  |  |
| Nonsmoker | Reference |  |
| Current/past smoker | 1.04 (1.02–1.06) | < 0.001 |
| GCS on admission | 1.00 (0.99–1.00) | 0.513 |
| Barthel index on admission |  |  |
| 0 | 1.14 (1.09–1.18) | < 0.001 |
| 5–35 | 1.04 (1.01–1.08) | 0.024 |
| 40–55 | 0.97 (0.93–1.02) | 0.273 |
| 60–95 | 0.99 (0.95–1.04) | 0.803 |
| 100 | Reference |  |
| Charlson comorbidity index | 1.04 (1.03–1.05) | < 0.001 |
| Pneumonia severity score | 1.14 (1.12–1.15) | < 0.001 |
| Risk factors for antibiotic-resistant pathogens |  |  |
| 0 | Reference |  |
| 1–2 | 1.05 (1.01–1.09) | 0.007 |
| ≥3 | 1.09 (1.02–1.18) | 0.019 |
| ICU admission | 1.46 (1.35–1.57) | < 0.001 |
| HCU admission | 1.38 (1.33–1.45) | < 0.001 |
| Admission from nursing home | 1.21 (1.18–1.24) | < 0.001 |
| Ambulance transport | 0.93 (0.91–0.95) | < 0.001 |
| Fiscal year |  |  |
| 2014–2015 | Reference |  |
| 2016–2017 | 0.90 (0.88–0.92) | < 0.001 |
| 2018–2019 | 0.89 (0.86–0.91) | < 0.001 |
| 2020–2021 | 0.94 (0.91–0.96) | < 0.001 |
| Aspiration pneumonia | 0.82 (0.79–0.84) | < 0.001 |
| History of tracheostomy | 0.86 (0.64–1.14) | 0.295 |
| Hypertension | 0.92 (0.90–0.94) | < 0.001 |
| Diabetes mellitus | 1.04 (1.02–1.07) | 0.001 |
| Dyslipidaemia | 0.95 (0.92–0.99) | 0.006 |
| Lung disease |  |  |
| Chronic obstructive pulmonary disease | 1.08 (1.04–1.13) | < 0.001 |
| Interstitial Pneumonia | 1.73 (1.64–1.81) | < 0.001 |
| Bronchiectasis & NTM of the lungs | 2.56 (2.40–2.74) | < 0.001 |
| Fungal lung disease | 2.15 (1.85–2.49) | < 0.001 |
| Lung cancers | 1.23 (1.18–1.30) | < 0.001 |
| Chronic respiratory failure | 1.18 (1.12–1.25) | < 0.001 |
| Oesophageal disorders and dysphagia | 0.94 (0.90–0.98) | 0.004 |
| Cerebrovascular disease | 0.93 (0.91–0.96) | < 0.001 |
| Neurologic disease | 0.90 (0.86–0.95) | < 0.001 |
| Cardiovascular disease | 0.89 (0.86–0.92) | < 0.001 |
| Liver disease | 0.97 (0.92–1.03) | 0.285 |
| Chronic kidney failure | 0.96 (0.92–1.00) | 0.060 |
| Immunodeficiency disorders | 1.42 (1.09–1.84) | 0.010 |
| Haematological malignancy | 2.22 (2.06–2.39) | < 0.001 |
| Non-haematological malignancy | 1.00 (0.95–1.05) | 0.937 |
| Solid organ transplantation | 1.25 (1.00–1.57) | 0.047 |
| Dementia | 0.82 (0.80–0.85) | < 0.001 |
| Factors prior to 90 days of admission |  |  |
| Hospitalisation | 1.08 (1.05–1.12) | < 0.001 |
| Antibiotic use | 1.25 (1.21–1.30) | < 0.001 |
| Immunosuppressive therapy | 1.34 (1.23–1.46) | < 0.001 |
| Steroid | 1.21 (1.16–1.26) | < 0.001 |
| Chemotherapy | 1.64 (1.56–1.72) | < 0.001 |
| Nutrition within 2 days of admission |  |  |
| Oral feeding | 0.66 (0.64–0.67) | < 0.001 |
| Tube feeding | 1.18 (1.11–1.26) | < 0.001 |
| Total parenteral nutrition | 1.29 (1.16–1.43) | < 0.001 |
| Treatment within 2 days of admission |  |  |
| Oxygenation | 1.25 (1.22–1.27) | < 0.001 |
| Mechanical ventilation | 1.66 (1.58–1.75) | < 0.001 |
| Vasopressors | 1.90 (1.80–2.00) | < 0.001 |
| Renal replacement therapy | 1.42 (1.32–1.53) | < 0.001 |
| Tetracyclines | 1.28 (1.18–1.39) | < 0.001 |
| Macrolides | 0.98 (0.95–1.02) | 0.280 |
| Quinolones | 1.67 (1.59–1.76) | < 0.001 |
| Anti-MRSA antibiotics | 4.21 (3.75–4.72) | < 0.001 |
| Steroids | 1.48 (1.42–1.55) | < 0.001 |
| Immunosuppressants | 1.25 (1.12–1.40) | < 0.001 |
| Proton pump inhibitors | 1.18 (1.16–1.21) | < 0.001 |
| Hypnotics | 0.97 (0.94–0.99) | 0.005 |
| Antipsychotics | 0.93 (0.91–0.96) | < 0.001 |
| Teaching hospital admission | 0.90 (0.88–0.91) | < 0.001 |

**Supplementary Table 4. Second-stage instrumental variable regression results.**

| Variables | Odds ratio  (95% Confidence interval) | *P* |
| --- | --- | --- |
| Broad-spectrum antibiotics | 1.01 (0.92–1.10) | 0.900 |
| Residuals | 1.29 (1.17–1.42) | < 0.001 |
| Age | 1.02 (1.02–1.02) | < 0.001 |
| Male | 1.41 (1.37–1.45) | < 0.001 |
| BMI, kg/m^2^ |  |  |
| <18.50 | 1.29 (1.24–1.35) | < 0.001 |
| 18.50–24.99 | Reference |  |
| 25.00–29.99 | 0.85 (0.80–0.89) | < 0.001 |
| ≥30.00 | 0.76 (0.67–0.85) | < 0.001 |
| Smoking history |  |  |
| Nonsmoker | Reference |  |
| Current/past smoker | 0.95 (0.92–0.98) | 0.004 |
| GCS on admission | 0.96 (0.96–0.97) | < 0.001 |
| Barthel index on admission |  |  |
| 0 | 2.97 (2.76–3.21) | < 0.001 |
| 5–35 | 2.04 (1.89–2.21) | < 0.001 |
| 40–55 | 1.72 (1.57–1.89) | < 0.001 |
| 60–95 | 1.27 (1.15–1.39) | < 0.001 |
| 100 | Reference |  |
| Charlson comorbidity index | 1.14 (1.11–1.16) | < 0.001 |
| Pneumonia severity score | 1.48 (1.46–1.50) | < 0.001 |
| Risk factors for antibiotic-resistant pathogens |  |  |
| 0 | Reference |  |
| 1–2 | 1.11 (1.06–1.17) | < 0.001 |
| ≥3 | 1.18 (1.05–1.32) | 0.005 |
| ICU admission | 0.76 (0.69–0.84) | < 0.001 |
| HCU admission | 0.98 (0.92–1.03) | 0.402 |
| Admission from nursing home | 1.09 (1.06–1.12) | < 0.001 |
| Ambulance transport | 1.03 (1.00–1.05) | 0.087 |
| Fiscal year |  |  |
| 2014–2015 | Reference |  |
| 2016–2017 | 1.03 (1.00–1.07) | 0.083 |
| 2018–2019 | 1.01 (0.97–1.05) | 0.593 |
| 2020–2021 | 1.25 (1.21–1.30) | < 0.001 |
| Aspiration pneumonia | 0.75 (0.72–0.78) | < 0.001 |
| History of tracheostomy | 0.38 (0.20–0.71) | 0.003 |
| Hypertension | 0.64 (0.62–0.66) | < 0.001 |
| Diabetes mellitus | 0.86 (0.83–0.89) | < 0.001 |
| Dyslipidaemia | 0.78 (0.73–0.82) | < 0.001 |
| Lung disease |  |  |
| Chronic obstructive pulmonary disease | 0.68 (0.64–0.72) | < 0.001 |
| Interstitial Pneumonia | 2.06 (1.93–2.20) | < 0.001 |
| Bronchiectasis & NTM of the lungs | 1.33 (1.19–1.47) | < 0.001 |
| Fungal lung disease | 1.69 (1.36–2.10) | < 0.001 |
| Lung cancers | 1.88 (1.75–2.01) | < 0.001 |
| Chronic respiratory failure | 1.07 (1.00–1.16) | 0.067 |
| Oesophageal disorders and dysphagia | 0.78 (0.74–0.83) | < 0.001 |
| Cerebrovascular disease | 0.76 (0.73–0.78) | < 0.001 |
| Neurologic disease | 0.73 (0.67–0.79) | < 0.001 |
| Cardiovascular disease | 1.02 (0.97–1.07) | 0.413 |
| Liver disease | 0.95 (0.88–1.04) | 0.251 |
| Chronic kidney failure | 1.22 (1.16–1.29) | < 0.001 |
| Immunodeficiency disorders | 0.84 (0.49–1.44) | 0.533 |
| Haematological malignancy | 1.41 (1.26–1.58) | < 0.001 |
| Non-haematological malignancy | 1.18 (1.11–1.26) | < 0.001 |
| Solid organ transplantation | 0.45 (0.20–1.00) | 0.050 |
| Dementia | 0.54 (0.52–0.57) | < 0.001 |
| Factors prior to 90 days of admission |  |  |
| Hospitalisation | 1.24 (1.17–1.31) | < 0.001 |
| Antibiotic use | 1.01 (0.96–1.08) | 0.659 |
| Immunosuppressive therapy | 0.98 (0.83–1.17) | 0.826 |
| Steroid | 1.00 (0.94–1.08) | 0.897 |
| Chemotherapy | 1.22 (1.12–1.33) | < 0.001 |
| Nutrition within 2 days of admission |  |  |
| Oral feeding | 0.58 (0.57–0.60) | < 0.001 |
| Tube feeding | 0.92 (0.84–1.01) | 0.077 |
| Total parenteral nutrition | 1.18 (1.03–1.34) | 0.016 |
| Treatment within 2 days of admission |  |  |
| Oxygenation | 1.88 (1.82–1.95) | < 0.001 |
| Mechanical ventilation | 1.98 (1.85–2.12) | < 0.001 |
| Vasopressors | 1.35 (1.27–1.44) | < 0.001 |
| Renal replacement therapy | 1.01 (0.90–1.13) | 0.841 |
| Tetracyclines | 1.25 (1.11–1.40) | < 0.001 |
| Macrolides | 1.00 (0.95–1.05) | 0.932 |
| Quinolones | 1.19 (1.10–1.28) | < 0.001 |
| Anti-MRSA antibiotics | 1.16 (1.01–1.34) | 0.035 |
| Steroids | 1.45 (1.37–1.53) | < 0.001 |
| Immunosuppressants | 1.00 (0.82–1.24) | 0.964 |
| Proton pump inhibitors | 0.97 (0.94–1.00) | 0.065 |
| Hypnotics | 0.86 (0.83–0.90) | < 0.001 |
| Antipsychotics | 1.16 (1.12–1.21) | < 0.001 |
| Teaching hospital admission | 0.91 (0.88–0.93) | < 0.001 |

**Supplementary Table 5. Sensitivity analysis of outcomes between the two groups**

|  | Narrow-spectrum group | Broad-spectrum group | Risk difference | 95% confidence interval | *P* |
| --- | --- | --- | --- | --- | --- |
| **Patients who did not receive combination therapy (%)**  **[Narrow (n = 435,836) vs. Broad (n = 141,707)]** |  |  |  |  |  |
| Primary outcome (%) |  |  |  |  |  |
| 30 day in-hospital mortality | 9.9% | 10.0% | 0.1% | −0.6% to 0.9% | 0.734 |
|  |  |  |  |  |  |
| **Patients from nursing or other long-term care facilities (%)**  **[Narrow (n = 161,733) vs. Broad (n = 49,941)]** |  |  |  |  |  |
| Primary outcome (%) |  |  |  |  |  |
| 30 day in-hospital mortality | 11.2% | 11.3% | 0.1% | −1.3% to 1.6% | 0.836 |
|  |  |  |  |  |  |
| **An analysis excluding underweight as a risk factor for antibiotic-resistant pathogens (%)** |  |  |  |  |  |
| Primary outcome (%) |  |  |  |  |  |
| 30 day in-hospital mortality | 9.9% | 10.0% | 0.1% | −0.7% to 0.8% | 0.853 |
